# Supplementary material for: Cyanidin-3-O-Glucoside Rescues Zearalenone-Induced Apoptosis via the ITGA7-PI3K-AKT Signaling Pathway in Porcine Ovarian Granulosa Cells
Source: Int J Mol Sci. 2023 Feb 23;24(5):4441. doi: 10.3390/ijms24054441 (PMC10002597; doi:10.3390/ijms24054441)
Supplement: Supplementary file 1 [file ijms-24-04441-s001.zip › ijms-2198651-supplementary.pdf]

Supporting Information

Cyanidin-3-O-glucoside Rescues Zearalenone-induced Apoptosis via the ITGA7-PI3K-AKT Signaling Pathway in Porcine Ovarian Granulosa Cells

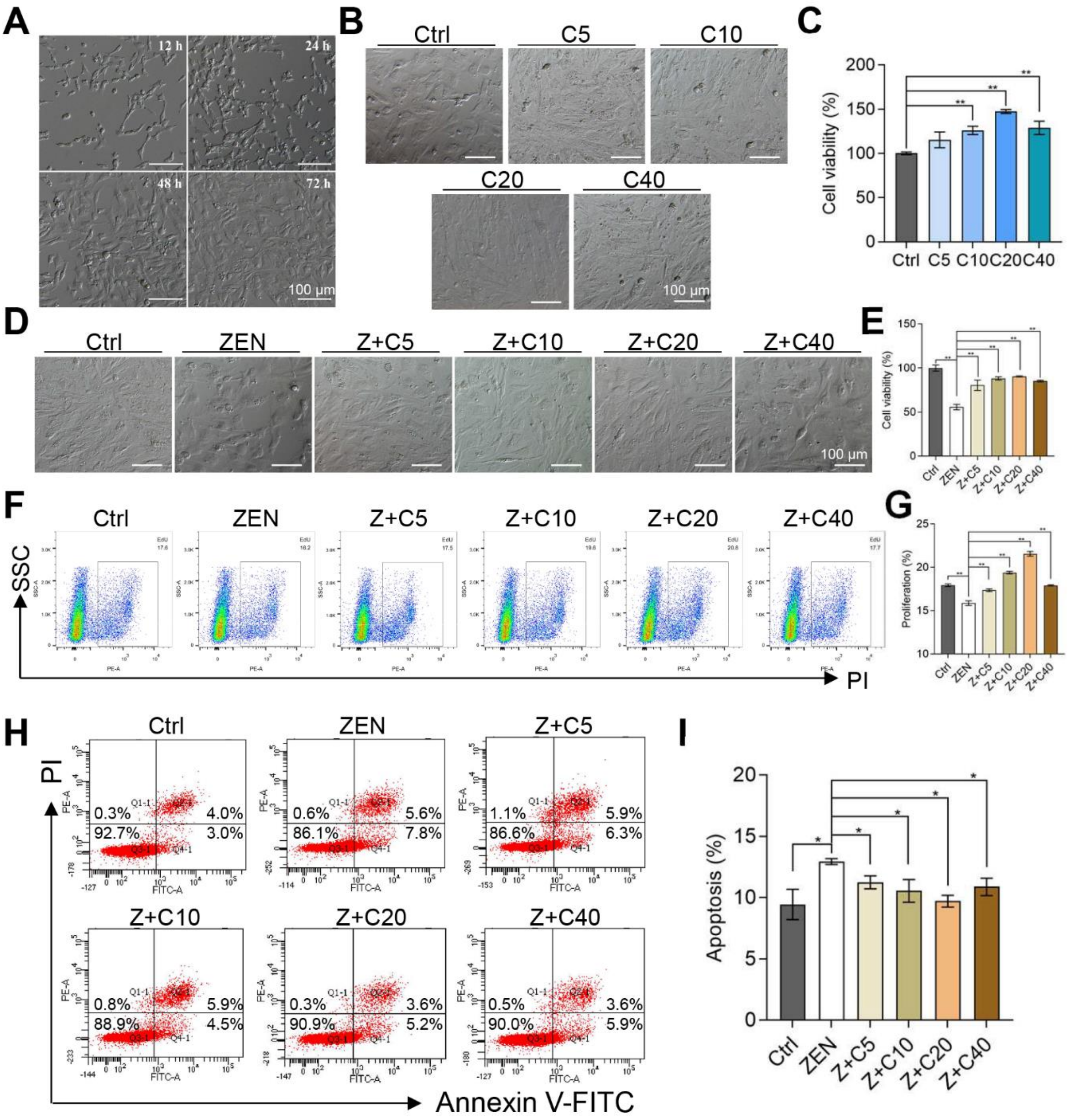

Figure S1. 20 μM is the optimum concentration of C3G to protect pGCs from toxic effects induced by ZEN**.** (A) Morphology of primary pGCs after cultivation for 12 h, 24 h, 48 h and 72 h, respectively. (B) pGCs were harvested at 24 h after addition with 5 μM, 10 μM, 20 μM, 40 μM C3G, respectively. The corresponding concentration of DMSO as a vehicle control. (C) Representative results of cell viability detected by CCK-8 determined from images in (B). (D) pGCs were collected at 24 h after addition of 30 μM ZEN and/or 5 μM, 10 μM, 20 μM, 40 μM C3G, respectively. The corresponding concentration of DMSO as a vehicle control. (E) Representative results of cell viability detected by CCK-8 determined from images in (D). (F) The proliferation rates were detected by flow cytometry using EdU staining in the six groups. (G) Representative results of cell proliferation rates determined from flow cytometry in (F). (H) The apoptosis were detected by flow cytometry using annexin V-FITC/PI staining. (I) Representative results of apoptosis cell rates determined from (H). Results were quantified as mean ± SEM; n = 3. All experiments were repeated at least three times. $^{*}p < 0.05$, $^{**}p < 0.01$. Scale bar: 100 μm.

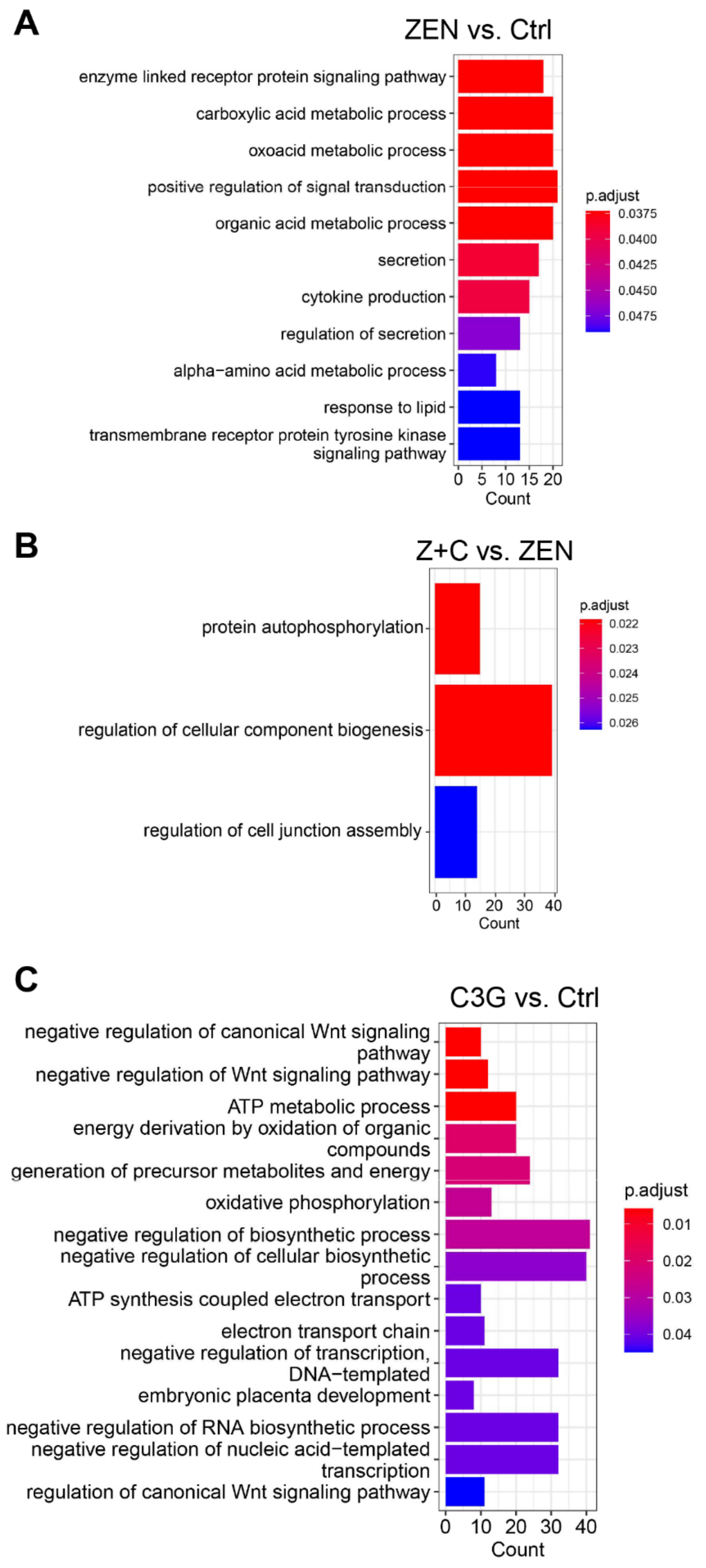

Figure S2. GO enrichment analysis. The histogram demonstrates GO enrichment results of DEmRNAs from ZEN versus Ctrl (A), Z+C versus ZEN (B) and C3G versus Ctrl (C) groups.

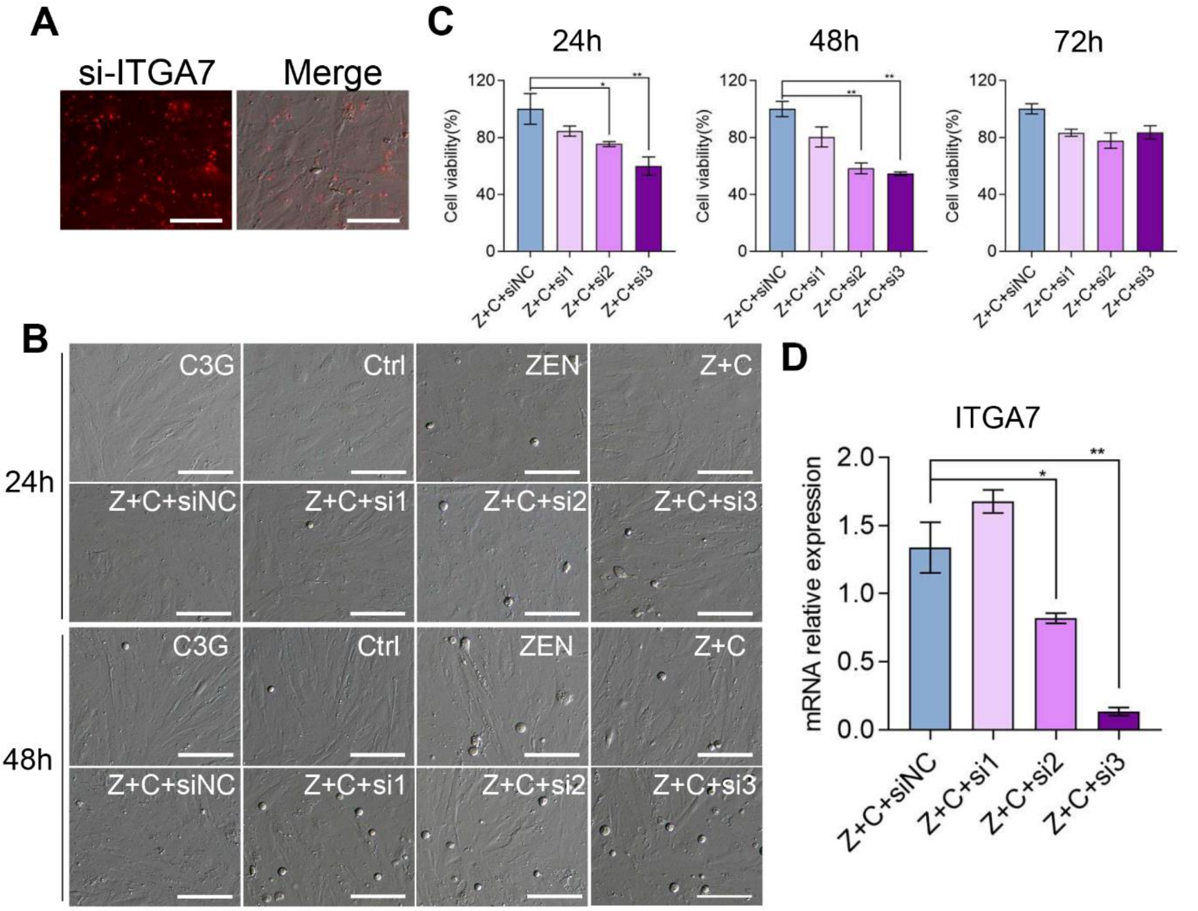

Figure S3. siITGA7 exploration and optimization of transfection conditions. (A) Morphology of primary pGCs transfected with si-ITGA7 (red) fragments for 6 h. (B) pGCs were collected at 24 h and 48 h in the Ctrl, ZEN, Z+C, C3G, NC, Z+C+si1, Z+C+si2, Z+C+si3 groups, respectively. (C) Cell viability of pGCs harvested at 24 h, 48 h and 72 h after transfection with ITGA7-Sus-1282 (si1), ITGA7-Sus-1731 (si2), and ITGA7-Sus-3018 (si3) fragments, respectively. (D) Relative mRNA expression levels of *ITGA7* in pGCs transfected with si1, si2, and si3 fragments. Results were quantified as mean ± SEM; n = 3. All experiments were repeated at least three times. $^{*}p < 0.05$, $^{**}p < 0.01$. Scale bar: 100 μm.

Tables

Table S1 Sequences of mRNA Primers

Primer name	Pubmed ID	Sequence (5′→3′)	GC(%)	Tm (℃)
ATF4	NM_001123078.1	F: CGATTCCATCAAAACTCCGC	50.0	62.8
		R: CACTGACCAACCCATCCACA	55.0	62.9
CCND3	NM_001078678.1	F: TACACGGACCACTCTGTCTCTC	54.5	59.4
		R: TGGCAAAGGTGTAATCTGTAGC	45.5	59.3
CDKN1A	XM_013977858.2	F: AACTTTGAGGTCCCCTTGCC	55.0	60.18
		R: GGGCCCTACTTTCACTGGAC	60.0	60.04
GAPDH	NM_001206359.1	F: GCCATCACCATCTTCCAGG	57.9	61.5
		R: TCACGCCCATCACAAACAT	47.4	61.0
ITGA7	XM_021091233.1	F: AGACGGCTTCCCAGACATTG	55.0	60.4
		R: AATGGTTCCCATCCACGTCC	55.0	60.4
PCK2	NM_001161753.1	F: CAAGGATGTGGCACGAGTAGA	52.4	61.2
		R: CACCACATAGGCTGAGTCCG	60.0	61.7

Table S2 Antibodies uesd in the experiment of WB

Names	Description	Company	Catalogue#	Dilution
AKT	Rabbit polyclonal	Sangong	D15537	500×
p-AKT(s473)	Rabbit polyclonal	Affinity	AF0016	500×
CCND3	Rabbit polyclonal	Affinity	DF6229	500×
CDKN1A	Rabbit polyclonal	Sangong	D120403	200×
β-actin	Rabbit polyclonal	Sangong	D110001	1000×
ITGA7	Rabbit polyclonal	ABcolonal	A14246	500×
PIK3CA	Rabbit polyclonal	ABcolonal	A0265	1000×
p-PI3K(p85)	Rabbit polyclonal	ABcolonal	A11177	1000×
Caspase-9	Rabbit polyclonal	Abcam	ab202068	1000×
PCNA	Mouse polyclonal	Abcam	ab29	1000×
Bcl-2	Rabbit polyclonal	Beyotime	AB112-1	1000×
Bax	Rabbit polyclonal	Cell signaling Technology(CST)	2772S	1000×
HRP-IgG	Goat anti Rabbit	Beyotime	A0208	2000×
HRP-IgG	Goat anti mouse	Beyotime	A0216	2000×

Table S3 Consequences of si-*ITGA7*

Names	Sequence (5′→3′)
Negtive control (NC)	Sense: UUCUCCGAACGUGUCACGUTT
	Antisense: ACGUGACACGUUCGGAGAATT
ITGA7-Sus-1282 (si1)	Sense: GGCCCUGAAUAGCUACUUATT
	Antisense: UAAGUAGCUAUUCAGGGCCTT
ITGA7-Sus-1731 (si2)	Sense: GGGAUGGGAAAGUCUUCAUTT
	Antisense: AUGAAGACUUUCCCAUCCCTT
ITGA7-Sus-3018 (si3)	Sense: GCAGCAAGGUCAAGUAUGATT
	Antisense: UCAUACUUGACCUUGCUGCTT

Table S4 Analysis of BP GO terms of DEmRNAs in the ZEN versus Ctrl group

ID	Description	GeneRatio	P_{adjust}
GO:0007167	enzyme linked receptor protein signaling pathway	18/160	0.029728
GO:0019752	carboxylic acid metabolic process	20/160	0.029728
GO:0033993	response to lipid	14/160	0.029728
GO:0043436	oxoacid metabolic process	20/160	0.029728
GO:0009967	positive regulation of signal transduction	21/160	0.029728
GO:0006082	organic acid metabolic process	20/160	0.029728
GO:0046903	secretion	17/160	0.029728
GO:0051091	positive regulation of DNA-binding transcription factor activity	9/160	0.029728
GO:0001816	cytokine production	15/160	0.030848
GO:0051046	regulation of secretion	13/160	0.035444
GO:0051049	regulation of transport	23/160	0.035444
GO:1901605	alpha-amino acid metabolic process	8/160	0.036786
GO:0007169	transmembrane receptor protein tyrosine kinase signaling pathway	13/160	0.041688
GO:0022612	gland morphogenesis	6/160	0.044479
GO:0051090	regulation of DNA-binding transcription factor activity	10/160	0.044479
GO:0003008	system process	23/160	0.044479
GO:0007267	cell-cell signaling	21/160	0.044479
GO:0010647	positive regulation of cell communication	21/160	0.044479
GO:0030282	bone mineralization	6/160	0.044479
GO:0051047	positive regulation of secretion	8/160	0.044479
GO:0030111	regulation of Wnt signaling pathway	8/160	0.044645
GO:0023056	positive regulation of signaling	21/160	0.044645
GO:0048584	positive regulation of response to stimulus	25/160	0.046164
GO:0016055	Wnt signaling pathway	10/160	0.046164
GO:0140352	export from cell	15/160	0.046164
GO:0051050	positive regulation of transport	13/160	0.046164
GO:0001666	response to hypoxia	6/160	0.046164
GO:0044283	small molecule biosynthetic process	13/160	0.046164
GO:0198738	cell-cell signaling by wnt	10/160	0.046164
GO:0036293	response to decreased oxygen levels	6/160	0.048209

Table S5 Analysis of BP GO terms of DEmRNAs in the Z+C versus ZEN group

ID	Description	GeneRatio	P_{adjust}
GO:0046777	protein autophosphorylation	15/506	0.021827004
GO:0044087	regulation of cellular component biogenesis	39/506	0.021827004
GO:1901888	regulation of cell junction assembly	14/506	0.026274854

Table S6 Analysis of BP GO terms of DEmRNAs in the C3G versus Ctrl group

ID	Description	GeneRatio	P_{adjust}
GO:0090090	negative regulation of canonical Wnt signaling pathway	10/417	0.004305
GO:0030178	negative regulation of Wnt signaling pathway	12/417	0.004305
GO:0046034	ATP metabolic process	20/417	0.004305
GO:0015980	energy derivation by oxidation of organic compounds	20/417	0.014629
GO:0006091	generation of precursor metabolites and energy	24/417	0.015724
GO:0006119	oxidative phosphorylation	13/417	0.02181
GO:0009890	negative regulation of biosynthetic process	40/417	0.038149
GO:0042773	ATP synthesis coupled electron transport	10/417	0.039565
GO:0022900	electron transport chain	11/417	0.040567
GO:0031327	negative regulation of cellular biosynthetic process	39/417	0.040567
GO:0001892	embryonic placenta development	8/417	0.042338
GO:0060828	regulation of canonical Wnt signaling pathway	11/417	0.04672

Table S7 Analysis of KEGG terms of DEmRNAs in the ZEN versus Ctrl group

ID	Description	GeneRatio	P_{adjust}
ssc04020	Calcium signaling pathway	17/189	0.002609472
ssc01230	Biosynthesis of amino acids	9/189	0.002609472
ssc05146	Amoebiasis	10/189	0.003978355
ssc05205	Proteoglycans in cancer	14/189	0.008092439
ssc04151	PI3K-Akt signaling pathway	18/189	0.022021458

Table S8 Analysis of KEGG terms of DEmRNAs in the Z+C versus ZEN group

ID	Description	GeneRatio	P_{adjust}
ssc04510	Focal adhesion	38/606	1.75E-06
ssc04512	ECM-receptor interaction	23/606	1.94E-06
ssc05165	Human papillomavirus infection	51/606	1.94E-06
ssc01521	EGFR tyrosine kinase inhibitor resistance	18/606	0.000283245
ssc04151	PI3K-Akt signaling pathway	45/606	0.001276368
ssc04810	Regulation of actin cytoskeleton	32/606	0.001406276
ssc05230	Central carbon metabolism in cancer	15/606	0.001533879
ssc05222	Small cell lung cancer	18/606	0.002114752
ssc05205	Proteoglycans in cancer	30/606	0.002114752
ssc05146	Amoebiasis	18/606	0.003230987
ssc04210	Apoptosis	22/606	0.003230987
ssc04910	Insulin signaling pathway	22/606	0.003230987
ssc05412	Arrhythmogenic right ventricular cardiomyopathy	15/606	0.003821689
ssc04010	MAPK signaling pathway	36/606	0.006877165
ssc01522	Endocrine resistance	16/606	0.010423627
ssc04330	Notch signaling pathway	12/606	0.010423627
ssc04360	Axon guidance	25/606	0.011168897
ssc05215	Prostate cancer	16/606	0.012383725
ssc01250	Biosynthesis of nucleotide sugars	9/606	0.012383725
ssc05010	Alzheimer disease	43/606	0.012383725
ssc00052	Galactose metabolism	8/606	0.012383725
ssc05145	Toxoplasmosis	17/606	0.012383725
ssc04520	Adherens junction	13/606	0.012383725
ssc00051	Fructose and mannose metabolism	8/606	0.014357043
ssc04919	Thyroid hormone signaling pathway	18/606	0.01531653
ssc04922	Glucagon signaling pathway	16/606	0.01531653
ssc05221	Acute myeloid leukemia	12/606	0.017523406

Table S9 Analysis of KEGG terms of DEmRNAs in the C3G versus Ctrl group

ID	Description	GeneRatio	P_{adjust}
ssc05165	Human papillomavirus infection	38/477	0.001622
ssc05205	Proteoglycans in cancer	26/477	0.004568
ssc04714	Thermogenesis	28/477	0.004568
ssc05415	Diabetic cardiomyopathy	25/477	0.007344
ssc05135	Yersinia infection	19/477	0.011966
ssc04668	TNF signaling pathway	16/477	0.011966
ssc04922	Glucagon signaling pathway	15/477	0.012528
ssc04910	Insulin signaling pathway	18/477	0.012528
ssc04010	MAPK signaling pathway	30/477	0.012739
ssc05225	Hepatocellular carcinoma	20/477	0.013225
ssc00760	Nicotinate and nicotinamide metabolism	8/477	0.014016
ssc05215	Prostate cancer	14/477	0.014047
ssc05221	Acute myeloid leukemia	11/477	0.015646
ssc05224	Breast cancer	18/477	0.015646
ssc04390	Hippo signaling pathway	18/477	0.031781
ssc04152	AMPK signaling pathway	15/477	0.035217
ssc04611	Platelet activation	15/477	0.035217
ssc04931	Insulin resistance	14/477	0.036151
ssc05226	Gastric cancer	17/477	0.03676
ssc04657	IL-17 signaling pathway	12/477	0.03676
ssc05210	Colorectal cancer	12/477	0.03676
ssc04933	AGE-RAGE signaling pathway in diabetic complications	13/477	0.03676
ssc05230	Central carbon metabolism in cancer	10/477	0.03676
ssc03015	mRNA surveillance pathway	12/477	0.040749
ssc05213	Endometrial cancer	9/477	0.041415
ssc04935	Growth hormone synthesis, secretion and action	14/477	0.044474
ssc04550	Signaling pathways regulating pluripotency of stem cells	16/477	0.044622
ssc04071	Sphingolipid signaling pathway	14/477	0.044622
ssc04917	Prolactin signaling pathway	10/477	0.044825
ssc04261	Adrenergic signaling in cardiomyocytes	16/477	0.045633
ssc04919	Thyroid hormone signaling pathway	14/477	0.045633
ssc05010	Alzheimer disease	33/477	0.045633
ssc05208	Chemical carcinogenesis - reactive oxygen species	22/477	0.046684

Table S10 FPKM values and relative mRNA levels of 5 key genes in the PI3K-AKT signaling pathway

Genes / Group	*CDKN1A*		*CCND3*		*PCK2*		*ATF4*		*ITGA7*	
	FPKM	mRNA	FPKM	mRNA	FPKM	mRNA	FPKM	mRNA	FPKM	mRNA
Ctrl	80301.15±7938.78	1.02±0.14	28920.66±3049.19	1.02±0.14	11658.23±160.31	1.00±0.01	95632.20±1760.77	1.00±0.06	480.91±95.19	1.04±0.19
ZEN	214381.69±4996.54**	11.46±1.77**	39113.04±1237.54**	1.91±0.24**	29879.71±531.62**	3.71±0.08**	128334.81±3181.66**	1.23±0.07*	129.31±15.47**	0.39±0.05*
Z+C	126192.85±25323.42$^{\#\#}$	2.68±0.71$^{\#\#}$	24824.19±1991.07$^{\#\#}$	0.93±0.02$^{\#\#}$	15122.27±1037.70$^{\#\#}$	2.38±0.05$^{\#\#}$	79533.00±7365.08$^{\#\#}$	0.45±0.04$^{\#\#}$	737.50±151.67$^{\#\#}$	1.16±0.19$^{\#\#}$
C3G	48784.48±2391.17	2.19±0.36	13359.5385±417.92**	0.77±0.07	6700.52±542.87**	0.72±0.04**	80174.33±210.17*	0.33±0.04**	1471.83±98.31	1.79±0.22*

Note: *CDKN1A*, cyclin-dependent kinase inhibitor 1; *CCND3*, cyclin-D3; *PCK2*, phosphoenolpyruvate carboxykinase 2; *ATF4*, activating transcription factor 4; *ITGA7*, integrin subunit alpha 7. $^{*}P < 0.05$ and $^{**}P < 0.01$ vs. Ctrl group; $^{\#\#}P < 0.01$ vs. ZEN group.
